# Root Filling

**Published:** 1904-06

**Authors:** W. F. Litch

**Affiliations:** Philadelphia.


### ROOT FILLING.

By W. F. Litch, Philadelphia.

My favorite root filling is gutta-percha, and where I do fill roots immediately after the removal of the pulp, as is sometimes necessary, I find it an excellent plan to carry on a probe a small portion of powdered iodoform as near as possible to the root apex and then pack the gutta-percha on that. Iodoform is, clinically, antiseptic as well as anæsthetic, and hence is an excellent dressing for the stump of a removed pulp. The advantage of applying the iodoform before introducing the gutta-percha, rather than with it, is that if mixed with gutta-percha and heated the iodoform is likely to be decomposed and some of its good qualities impaired.—*Brief.*

